# Malaria in Eritrean migrants newly arrived in seven European countries, 2011 to 2016

**DOI:** 10.2807/1560-7917.ES.2019.24.5.1800139

**Published:** 2019-01-31

**Authors:** Klara Sondén, Thierry Rolling, Andreas Wångdahl, Elsie Ydring, Sabine Vygen-Bonnet, Robert Kobbe, Johan Douhan, Ulf Hammar, Janneke Duijster, Brechje de Gier, Joanne Freedman, Nicole Gysin, Klaus Stark, Flora Stevens, Lasse Skafte Vestergaard, Anders Tegnell, Anna Färnert

**Affiliations:** 1 Department of Medicine Solna, Karolinska Institutet, Stockholm, Sweden; 2 Department of Infectious Diseases, Karolinska University Hospital, Stockholm, Sweden; 3 Division of Infectious Diseases, I. Department of Internal Medicine, University Medical Center Hamburg-Eppendorf, Hamburg, Germany; 4 Clinical Research Department, Bernhard-Nocht-Institute for Tropical Medicine, Hamburg, Germany; 5 Department of Infectious Diseases, Västmanland Hospital, Västerås, Sweden; 6 Public Health Agency of Sweden, Stockholm, Sweden; 7 Robert Koch Institute, Berlin, Germany; 8 Department of Pediatrics, University Medical Center Hamburg-Eppendorf, Hamburg, Germany; 9 Unit of Biostatistics, Department of Epidemiology, Institute for Environmental Medicine, Karolinska Institutet, Stockholm, Sweden; 10 Department for Early Warning and Surveillance Center for Epidemiology and Surveillance of Infectious Diseases, National Institute for Public Health and the Environment, Bilthoven, the Netherlands; 11 Public Health England, London, United Kingdom; 12 Federal Office of Public Health, Bern, Switzerland; 13 Statens Serum Institute, Copenhagen, Denmark

**Keywords:** travellers, migrants, malaria, plasmodium vivax, outbreak, relapse

## Abstract

Global migration has resulted in a large number of asylum applications in Europe. In 2014, clusters of *Plasmodium vivax* cases were reported among newly arrived Eritreans. This study aimed to assess malaria among Eritrean migrants in Europe from 2011 to 2016. We reviewed European migration numbers and malaria surveillance data for seven countries (Denmark, Germany, Netherlands, Norway, Sweden, Switzerland and the United Kingdom) which received 44,050 (94.3%) of 46,730 Eritreans seeking asylum in Europe in 2014. The overall number of malaria cases, predominantly *P. vivax*, increased significantly in 2014 compared to previous years, with the largest increases in Germany (44 *P. vivax* cases in 2013 vs 294 in 2014, p < 0.001) and Sweden (18 in 2013 vs 205 in 2014, p < 0.001). Overall, malaria incidence in Eritreans increased from 1–5 to 25 cases per 1,000, and was highest in male teenagers (50 cases/1,000). In conclusion, an exceptional increase of malaria cases occurred in Europe in 2014 and 2015, due to rising numbers of Eritreans with high incidence of *P. vivax* arriving in Europe. Our results demonstrate potential for rapid changes in imported malaria patterns, highlighting the need for improved awareness, surveillance efforts and timely healthcare in migrants.

## Introduction

Between 2010 and 2013, approximately 5,200 to 5,700 cases of imported malaria were reported annually in Europe, in both travellers and migrants [1]. Most cases were caused by *Plasmodium falciparum* acquired in sub-Saharan Africa. *Plasmodium vivax* was the second most common species, accounting for approximately 10% to 25% of cases and was mainly acquired in Asia [2,3]. Europe saw a large increase in asylum seekers from 2013 to 2015, including people from malaria-endemic countries. To what extent the patterns of imported malaria are affected by changing migration patterns has not yet been explored.

Sporadic reports of *P. vivax* cases among newly arrived Eritrean migrants were published in early 2014 [4-6] and a review of clinical records and national surveillance data in Sweden revealed a sudden increase in overall imported malaria cases, predominantly due to *P. vivax,* among newly arrived Eritreans, a group not previously noted for high numbers of imported malaria [4]. A similar increase in *P. vivax* cases between 2014 and 2015 was reported from Germany [5], Norway [6], the Netherlands [7], Denmark [8] and Switzerland [9].

The aim of this study was to establish the extent of malaria in Eritrean migrants in Europe in the period from 2011 to 2016. We performed an analysis of European migration data and analysed malaria surveillance data from the seven countries receiving the majority of Eritrean asylum applications in Europe during 2014 and 2015. In addition, we performed a retrospective review of clinical data from Germany and Sweden.

## Methods

### Migration data

Data on asylum applications from Eritrean citizens in 32 European countries between 2011 and 2016 were retrieved from the Eurostat database [10]. This included 28 European Union countries as well as the European Free Trade Agreement (EFTA) countries Iceland, Liechtenstein, Norway and Switzerland. In addition, weekly data on Eritrean asylum applications in Sweden from 2011 to 2016 were available at the Swedish Migration Agency in Sweden.

### Malaria surveillance data

Public health agencies in the seven countries (Denmark, Germany, The Netherlands, Norway, Sweden, Switzerland, and the United Kingdom (UK)) reporting > 2000 asylum applications from Eritrean citizens in 2014 were contacted with a request for malaria surveillance data from 2011 to 2016. The requested data included overall number of malaria cases and cases among Eritrean migrants by *Plasmodium* species and available data on patient origin with definitions of newly arrived Eritreans described in the various countries, (Table 1). The agencies included were Statens Serum Institute (SSI) in Denmark, Robert Koch Institute (RKI) in Germany, the Centre for Infectious Disease Control at the National Institute for Public Health and the Environment in the Netherlands (RIVM), the Norwegian Institute of Public Health, the Public Health Agency of Sweden, the Federal Office of Public Health in Switzerland, and Public Health England (PHE) in the UK.

**Table 1:** Eritrean asylum applications and available data for malaria cases in seven countries, Europe, 2011–2016

Year	Asylum applications from Eritrean citizens^a^						Malaria cases overall^b^				Malaria cases in Eritreans						
	Total^c^			Minors^d^			Total n	*P. vivax* n	*P. falciparum* n	Other^e^ n	Total n	Median a ge (IQR)	Sex (male)	*P. vivax* n	*P. falciparum* n	Other^e^ n	Rate (95% CI)^f^	
	n		%	n	%^g^													
Europe (EU and EFTA countries)																		
2011	10,430		100	2,530		24.3	NA	NA	NA	NA	NA	NA	NA	NA	NA	NA	NA	
2012	11,995		100	3,095		25.8	NA	NA	NA	NA	NA	NA	NA	NA	NA	NA	NA	
2013	20,300		100	3,605		17.8	NA	NA	NA	NA	46	NA	NA	NA	NA	NA	2.3 (1.4–3.7)	
2014	46,730		100	8,515		18.2	NA	NA	NA	NA	823	NA	NA	NA	NA	NA	17.6 (14.6–21.3)	
2015	47,025		100	12,120		25.8	NA	NA	NA	NA	686	NA	NA	NA	NA	NA	14.6 (12.1–17.6)	
2016	40,260		100	10,195		25.3	NA	NA	NA	NA	253	NA	NA	NA	NA	NA	6.2 (2.3–16.5)	
Denmark																		
2011	20		0.1	5		25.0	74	13	51	10	0	NA	NA	0	0	0	0.0 (0.0–184.4)	
2012	55		0.3	15		27.3	68	10	49	9	0	NA	NA	0	0	0	0.0 (0.0–67.1)	
2013	85		0.4	15		17.6	62	10	47	5	1	13	1/1	1	0	0	11.8 (0.3–65.5)	
2014	2,275		4.9	300		13.1	102	34	53	14	33	24 (21–27)	25/33	27	2	4	14.5 (10.0–20.4)	
2015	1,710		3.6	240		14.0	102	33	58	11	28	24 (21–25)	21/28	27	0	1	17.0 (11.4–24.4)	
2016	255		0.6	140		54.9	101	38	57	6	13	24 (17–26)	4/13	11	1	1	43.1 (32.6-58.4)	
Germany																		
2011	650	0.6		170		26.2	563	58	408	97	0	NA	NA	0	0	0	0.0 (0.0–5.7)	
2012	670	0.6		210		31.3	551	73	389	89	0	NA	NA	0	0	0	0.0 (0.0–5.5)	
2013	3,640	0.6		470		12.9	638	45	496	97	7	20 (17–25)	7/7	4	0	3	1.9 (0.8–4.0)	
2014	13,255	27.8		1,805		13.6	1,008	293	536	179	252	20 (17–24)	227/252	197	12	43	19.0 (16.7–21.5)	
2015	10,990	23.4		2,420		22.0	1,063	305	602	156	197	20 (17–24)	176/197	148	10	39	17.9 (15.5–20.6)	
2016	1,9100	47.4		4,025		21.1	960	168	663	129	55	19 (17–26	43/55	45	2	8	2.9 (2.2–3.8)	
The Netherlands																		
2011	500	4.8		65		13.0	242	33	175	35	1	53	1/1	1	0	0	2.0 (0.0–11.14)	
2012	480	4.0		75		15.6	199	30	142	27	2	29 (19–39)	2/2	1	1	0	4.1 (0.5–15.0)	
2013	920	4.5		110		12.0	166	16	128	22	4	36 (27–42)	4/4	2	1	1	4.3 (1.2–11.1)	
2014	3,910	8.2		770		19.7	282	84	163	38	99	20 (17–24)	85/98	77	4	18	25.3 (20.6–30.8)	
2015	7,455	15.9		1,730		23.2	344	129	179	36	118	22 (17–25)	85/118	97	8	13	15.8 (13.1–19.0)	
2016	1,925	4.8		960		49.9	250	60	168	22	46	17 (14–22)	27/46	42	1	3	23.9 (17.5–31.9)	
Norway																		
2011	1,255	12.0		215		17.1	31	6	20	5	0	33 (4–52)	2/3	0	0	0	0.0 (0.0–2.9)	
2012	1,185	9.9		185		15.6	37	6	26	5	3	33 (4–52)	2/3	1	2	0	2.5 (0.5–7.4)	
2013	3,250	16.0		385		11.8	87	16	53	18	13	23 (21–25)	11/13	8	1	4	4.0 (2.1–6.8)	
2014	2,880	6.0		475		16.5	120	58	49	13	53	20 (17–23)	43/53	47	1	5	18.4 (13.8-24.1)	
2015	2,950	6.3		870		29.5	93	54	35	4	35	20 (16–25)	30/35	31	2	2	11.9 (8.3-16.5)	
2016	590	1.5		115		19.5	75	48	16	9	7	26 (16–28)	4/7	4	1	2	11.9 (4.8-24.3)	
Sweden																		
2011	1,705	16.3		385		22.6	95	16	61	18	2	33 (24–41)	1/2	2	0	0	1.2 (0.1-4.2)	
2012	2,405	20.1		500		20.8	85	13	66	6	1	19	1/1	0	0	1	0.4 (0.01-2.3)	
2013	4,880	24.0		840		17.2	119	18	81	20	8	24 (19–31)	7/8	7	1	0	1.6 (0.7-3.2)	
2014	11,530	24.7		2,295		19.9	354	205	103	48	214	21 (17–25)	162/214	175	8	36 (16.8)	18.6 (16.2-21.2)	
2015	7,230	15.4		2,740		37.9	250	100	102	48	93	19 (17–24)	73/93	65	8	20 (21.5)	12.9 (10.4-15.8)	
2016	1,150	2.9		315		27.4	154	37	83	34	7	16 (15–25)	4/7	5	0	2 (28.6)	6.1 (2.4-12.5)	
Switzerland																		
2011	3,450	33.1		1,275		37.0	189	14	146	29	3	23 (17–31)	2/3	2	1	0	0.9 (0.2–2.5)	
2012	4,410	36.8		1,810		41.0	149	20	102	27	12	14 (8–23)	7/12	10	0	2	2.7 (1.4–4.7)	
2013	2,560	12.6		1,325		51.8	159	21	118	20	5	23(13–31)	3/5	5	0	0	1.9 (0.6–4.6)	
2014	6,920	14.5		2,055		29.7	316	113	152	51	105	20 (16–24)	90/105	81	6	18	15.2 (12.4–18.4)	
2015	9,965	21.2		3,075		30.9	435	157	200	78	151	20 (16–24)	120/151	110	8	33	15.3 (13.0–18.0)	
2016	5,178	12.9		2,873		55.5	316	64	198	54	46	18 (16–23)	32/54	33	1	12	8.9 (6.5–11.9)	
United Kingdom																		
2011	865	8.3		130		15.0	1,677	416	1,149	112	5	23 (19–48)	4/5	1	4	0	5.8 (1.9–13.5)	
2012	785	6.6		110		14.0	1,378	271	1,002	105	2	22	2/2	1	1	0	2.6 (0.3–9.2)	
2013	1,460	7.2		160		11.0	1,501	179	1,192	130	0	ND	ND	0	0	0	0.0 (0.0–2.5)	
2014	3,280	7.0		460		14.0	1,586	225	1,169	169	20	22 (19–24)	18/20	15	1	2	6.1 (3.7-9.4)	
2015	3,740	8.0		715		19.1	1,400	207	1,068	125	20	21 (18–25)	18/20	17	0	3	5.4 (3.3–8.3)	
2016	1,295	3.2		490		37.8	1,618	166	1,308	144	13	21 (17–26)	11/13	9	3	1	10.0 (5.4–17.2 )	

CI: confidence interval; EFTA: European Free Trade Association; EU: European Union; IQR: interquartile range; NA: not applicable; ND: no data available; *P. falciparum*: *Plasmodium falciparum*; *P. vivax*: *Plasmodium vivax*.

^a^ Data from [10].

^b^ Malaria cases in the national surveillance data.

^c^ Proportion of Eritrean asylum applicants in Europe.

^d^ Proportion of minors among Eritrean asylum applicants in the respective countries.

^e^ Other species, mixed infections and undefined species.

^f^ Incidence rate calculated as malaria cases in Eritreans per 1,000 asylum applications from Eritreans the corresponding year.

^g^ % of minors among the Eritrean asylum applicants

Malaria cases in Eritreans were defined as follows:

•      in Germany, Netherlands, Switzerland and United Kingdom, as people with Eritrea as country of birth and newly arrived entrants;

•      in Germany, people counted as Eritreans were also cases of malaria for whom country of origin was not indicated, but for whom date of arrival in Germany was 1 year or less prior to malaria diagnosis, and country of infection was indicated as ‘Eritrea’;

•      in Denmark, cases of malaria where Eritrea was given as country of infection;

•      in Sweden cases of malaria in refugees/asylum applicants from Eritrea and/or Eritrea as country of infection.

A more in-depth review was performed using surveillance and clinical data from Germany and Sweden, the two countries with the highest number of Eritrean asylum seekers.

### Clinical data

In addition to surveillance data, medical records of Eritreans diagnosed with malaria at adult and paediatric hospital units in Sweden (21 hospitals in 17 of 21 counties) and Hamburg, Germany, from 2014 and 2015 were reviewed for clinical data and migration history.

### Ethical statement

The clinical review was performed with permission from the Regional Ethical Review Board, Sweden (EPN 2009/1328-31/5, 2010/1080-32 and 2012/1155-32). In Germany the study was granted exemption according to local law by Ärztekammer Hamburg from requiring ethics approval because it was a retrospective analysis on anonymised patient data.

### Statistical analyses

Statistical analyses were performed using Stata version 13 (StataCorp, College Station, Texas, United States). Categorical data was compared using chi-squared or Fisher´s exact tests, and continuous data using Wilcoxon-Mann-Whitney test. The number of malaria cases among Eritrean asylum seekers was calculated per 1,000 Eritreans arriving in the corresponding year. All model-based comparisons were made using Poisson regressions. To compare the rate of malaria infections in different years within the group of Eritrean asylum seekers, we used the number of malaria cases among recently arrived Eritreans (as defined in the national surveillance data) as outcome and total number of recently arrived Eritreans (asylum applications) as exposure. Univariate models were used to relate different lags (0–20 weeks) of the weekly number of Eritrean asylum applications and *P. vivax* cases in Sweden from 2011 to 2016, defining the highest McFadden R-squared [11] as the strongest association for weekly number of asylum applications predicting the number of *P. vivax* cases. Haemoglobin levels were correlated with symptom duration by the Spearman correlation.

## Results

### Migration data

In 2014, 46,730 (7.1%) of the 662,165 asylum applications in Europe were submitted by Eritreans, a statistically significant increase compared to previous years (Table 1, Supplementary Figure S1 p < 0.001). Eritreans constituted the largest group of asylum applicants from sub-Saharan Africa in Europe in 2014, followed by citizens from Nigeria (n = 21,330, 3.2%) and Somalia (n = 20,155, 3.0%) (p < 0.001).

Seven countries received 44,050 (94.3%) of 46,730 Eritreans seeking asylum in Europe in 2014 (in descending order): Germany, Sweden, Switzerland, Netherlands, Norway, Denmark, and the UK (range: 2,275–13,255) (Table 1). The remaining 25 EU/EFTA countries reported a median of 10 applications (range: 0–820).

The number of Eritreans coming to Europe to seek asylum in 2015 and 2016 remained at similar levels: 47,025 (3.4%) of 1,392,655, and 40,260 (3.2%) of 1,255,600 asylum applications in Europe were from Eritrean citizens, respectively, and again mainly in the same seven countries (93.7%). A large proportion of Eritreans seeking asylum in Europe in 2014 and 2015 were minors aged < 18 years: 8,515 (18.2%) and 12,120 (25.8%), respectively (Table 1). A majority were males: in 2014 the proportion was 34,375 of 46,730 (73.5%) and in 2015 33,055 of 47,025 (70.3%).

### Total number of malaria cases

The total number of malaria cases notified in the seven countries increased markedly in 2014 (n=3,771) compared to previous years (2011: 2,871, 2012: 2,463 and 2013: 2,731), and remained high in 2015 (n=3,692). *P. vivax* was the main species accounting for the increase and the proportion of malaria cases diagnosed as *P. vivax* increased in 2014 (Table 1).

### Malaria cases in Eritreans

We estimated the incidence of malaria in Eritreans per 1,000 Eritrean asylum applications in the seven respective countries. The incidence rate increased notably in 2014 (18/1,000) and remained at a similar level during 2015 (15/1,000) (Table 1). During 2016, the overall incidence returned to a lower level (6/1,000), similar to that seen from 2011 to 2013. The increase was only seen for *P. vivax* in 2014 and 2015, whereas the *P. falciparum* incidence in Eritreans remained low (< 1/1,000) between 2011 and 2016 in all countries.

The overall mean incidence from the seven countries was then used to estimate the total number of malaria cases in Eritrean asylum applicants in Europe: 47 among 20,300 Eritrean applicants in 2013, 823 among 46,730 in 2014, 686 among 47,025 in 2015, and 253 among 40,260 Eritreans in 2016 (Table 1).

### Germany

**Figure 1:**
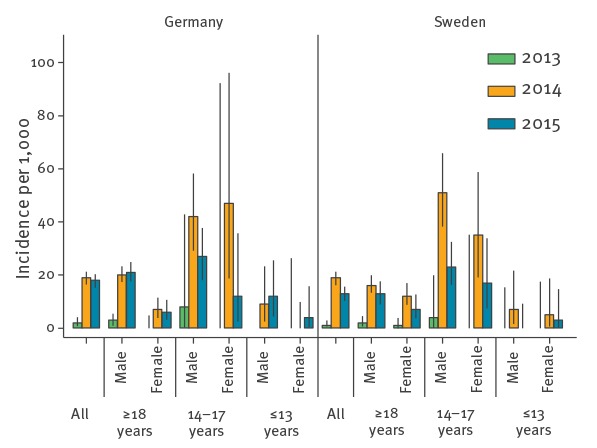
Incidence of malaria cases per 1,000 Eritrean asylum applicants in different age groups, Germany and Sweden, 2013–2015

Germany was the European country which received the highest number of asylum applications from Eritrea, with 13,255 in 2014, 10,990 in 2015 and 19,100 in 2016. The overall number of notified malaria cases, predominantly *P. vivax*, increased significantly in 2014 compared to previous years (44 *P. vivax* cases in 2013 versus 294 in 2014, p < 0.001). The incidence of malaria among Eritreans increased notably in 2014 with 19.0 cases per 1,000 (95% confidence interval (CI): 16.7–21.5) and remained high in 2015 with 17.9 (95% CI: 15.5–20.6). Cases were predominantly in male patients with 227 of 252 (90.1%) in 2014 and 176 of 197 (89.3%) in 2015. The median age was 20 years in 2014 and 2015. During 2016 the incidence was similar to the period prior to 2014 (Table 1). Malaria incidence in children (≤ 13 years) was 0 per 1,000 in 2013 and 2015, while teenagers aged 14 to 17 years had a high incidence in 2014 and 2015 (Figure 1). Year of arrival in Germany was available in the health surveillance data and 92% (95% CI: 89–95%) of malaria cases in Eritrean citizens in 2014 and 2015 were diagnosed in the year of arrival (Supplementary Table S2).

The black lines indicate the 95% confidence intervals.

The clinical presentation of malaria in Eritreans was assessed by reviewing clinical records of 66 patients at the University Medical Centre Hamburg-Eppendorf during 2014 and 2015. In total, 52 of 66 patients with ICD10 code B51 (*P. vivax* malaria), and none of the 130 patients with ICD10 code B50 (*P. falciparum* malaria) were Eritreans. Median age was 19 years (range: 12–52 years) and 44 patients (84.6%) were male. Patients reported a migration route starting in Eritrea and travelling through Ethiopia and/or Sudan, and then to Libya before crossing the Mediterranean Sea to Europe (Table 2). Some 38 (73.1%) patients reported that they had already been treated for malaria on the way to their destination country and one patient presented with severe malaria anaemia. The median symptom duration prior to contact with healthcare professionals in Hamburg was 3 days (range: 1–180) (Table 2). The proportion of *P. vivax* relapses after treatment in Germany was 7.8% (4/52).

**Table 2:** Clinical characteristics in Eritreans with malaria extracted from medical records in Sweden and Germany, 2014–2015 (n = 262)

Characteristics	Sweden (multicenter) N = 210	Germany (Hamburg) N = 52	p value
Age in years, median (range)	21 (2–61)	19 (12–52)	0.84
Proportion males	154/210	44/52	0.11
**Infection due to species of malaria**			
*Plasmodium vivax*	186	52	NA
*Plasmodium falciparum*	9	0	NA
*Plasmodium ovale*	9	0	NA
Mixed *Plasmodium vivax* and *Plasmodium falciparum*	6	0	NA
**Migration route from Eritrea**			
Via Sudan and Libya	68	21	0.08
Via Ethiopia, Sudan and Libya	97	14	NA
Missing data	45	17	NA
Migration duration in months, median (range)	4 (1–72)	6 (1–10)	0.56
**Malaria treatment during migration**			
Treated once	80	18	0.002
Treated 2–6 times	25	20	NA
Missing data	55	6	NA
**Clinical symptoms and laboratory findings**			
Symptom duration at diagnosis in days, median (range)	3 (0–180)	3 (1–180)	0.829
Haemogloblin g/L, median (range) (normal values 117-170)	114 (37–171)	125 (55–161)	0.004
Platelet count number x 10^9^/µl, median (range) ((normal values 150-400)	105 (5–289)	118 (23–380)	0.52
Severe malaria according to WHO criteria	20	1	0.07
Severe *Plasmodium vivax*	18	1	NA
Severe mixed infection (*Plasmodium falciparum* and *P. vivax*)	2	0	NA
Severe anaemia	11	1	NA
Other criteria for severe malaria	9	0	NA
Preventive anti-relapse treatment (primaquine) prescribed	173	51	1.000
Relapse after treatment	17	4	0.352

WHO: World Health Organization; NA: no analysis performed.

### Sweden

Sweden received 11,530 Eritrean asylum applications in 2014, 7,230 in 2015 and 1,150 in 2016 (Table 1). In Sweden, 119 malaria cases were reported in 2013 and 354 cases in 2014 (197% increase, p < 0.001). The increase was predominant for *P. vivax* (18 cases in 2013 versus 205 in 2014, p < 0.001). The incidence among Eritreans across age groups was estimated in Sweden from 2013 to 2015, with the highest estimated incidence in the 14–17-year age group with up to 50.7 cases per 1,000 Eritrean males (corresponding to 1 case/20 teenagers) in Sweden in 2014 (Figure 1). In 2015, the incidence in 14–17-year-olds declined, whereas it remained stable in adults.

**Figure 2:**
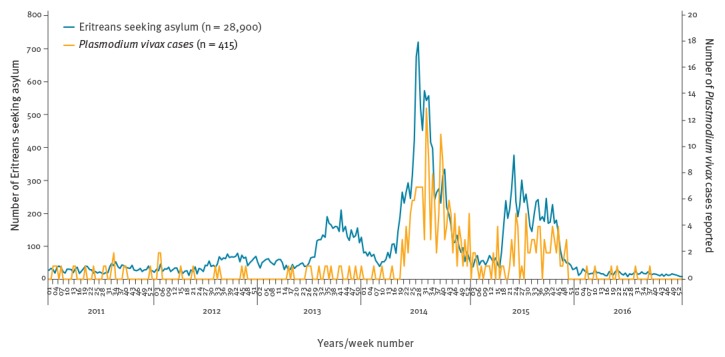
Weekly number of Eritreans seeking asylum and *Plasmodium vivax* cases reported in Sweden, 2011–2016

The weekly number of *P. vivax* cases in Sweden followed the number of Eritrean asylum applications, with a time lag of 5 weeks (McFadden’s R-squared: 0.31); and although the number of applications from Eritreans started to increase in 2013, *P. vivax* malaria cases did not rise before spring 2014 (Figure 2).

In Sweden, 210 medical records from 21 hospitals were reviewed, representing 69.7% of the 307 notified cases in newly arrived Eritreans during 2014 and 2015. The cases had a mean age of 21 years (range: 2–61) and 154 (72.9%) were males (Table 2). Twenty patients (9.5%) fulfilled the World Health Organization (WHO) criteria for severe malaria [12] (19 with *P. vivax* and two with *P. vivax* and *P. falciparum* mixed infection); and two were admitted to intensive care unit (one with vivax malaria and impaired consciousness and one with vivax malaria and severe anaemia). The most common sign of severe illness was severe anaemia (n = 12) with (haemogloblin level of 37–66 g/L (normal value: 117–164 g/L); and 20 patients with haemogloblin levels between 37 and 93 g/L received blood transfusions. Haemogloblin levels were inversely correlated with symptom duration (Spearman = −0.37, p < 0.001), and patients with severe malaria tended to have longer symptom duration (p = 0.087). Lower age was significantly associated with decreased haemogloblin levels (Spearman = 0.177, p = 0.0053), but not with symptom duration (Spearman = 0.01, p = 0.82), or severe malaria (Wilcoxon -Mann-Whitney test p = 0.9). The proportion of *P. vivax* relapses after treatment in Sweden was 8.1%.

**Figure 3:**
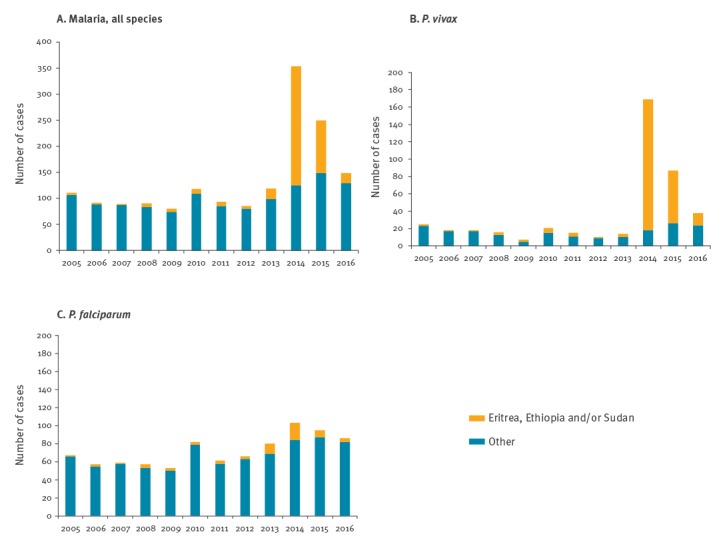
Number of (A) malaria, (B) *Plasmodium vivax* and (C) *Plasmodium falciparum* cases diagnosed in Sweden by reported possible country of infection, 2005–2016

Treatment for malaria along the route of travel to Sweden was reported in 105 (50%) of cases in Sweden (data were missing for 55 patients) and median symptom duration before healthcare contact in Sweden was 3 days (range: 0–180 days) (Table 2). Patients reported a migration route starting in Eritrea and travelling through Ethiopia and/or Sudan, and then to Libya before crossing the Mediterranean Sea to Europe (Table 2). These countries were reported as country of infection in the national malaria statistics in Sweden and accounted for the overall increase in 2014 and 2015 (Figure 3); only 10 of the 205 *P. vivax* cases in 2014 had other countries of infection (India, Pakistan, Afghanistan, Tanzania, Somalia, Peru and Papua New Guinea). When comparing individual data for 209 medical records from 2014 and 2015 with those stated in the surveillance data, the countries of infection matched in 62 (29.5%) patients, differed in 34 of 209, and were missing in the medical records of 105 patients (50.5%). Eight of 209 patients could not be identified in the national database. Hence, in 34 of the 96 patients with stated country of infection in both surveillance and medical record, the countries did not match.

*P*: *Plasmodium.*

Data based on patient-reported travel route and notifications in the medical record.

### Switzerland

Switzerland received between 6,920 and 9,965 annual Eritrean asylum applicants in 2014 to 2016. The total number of malaria cases approximately doubled during this time period compared to the period 2011 to 2013, and the increase was predominantly due to malaria in Eritreans (Table 1). This group represented 3 to 12 cases annually in 2011 to 2013 and 46 to 151 in the following 3 years. Malaria incidence was approximately 15 per 1,000 Eritreans in 2014 and 2015 but dropped to 8.8 in 2016, although 5,178 asylum applicants registered in the same year.

### The Netherlands

The Netherlands received between 1,925 and 7,455 asylum applications from Eritreans in 2014 to 2016, in parallel with a marked increase in the total number of malaria cases represented by *P. vivax,* whereas for other malaria species, no increase was seen. The median age in the group of Eritreans with *P. vivax* malaria was 20 years in the period 2014 to 2016 and male sex was more common than female. In contrary to most other countries, the Netherlands reported incidence rates similar to 2014 and 2015 also during 2016 with an incidence rate of 23.9 (17.5–31.9).

### Norway

Norway received approximately 3,000 Eritrean asylum applicants per year in the years 2013 to 2015. Despite similar numbers of asylum applications during these years, the increase in malaria cases did not occur until 2014. Analysis of malaria incidence rates revealed 4.0 (95% CI: 2.1–6.8), 18.4 (95% CI: 13.8–24.1) and 11.9 (95% CI: 8.3–16.5) per 1,000 Eritreans in 2013, 2014 and 2015, respectively. Data for 2016 as well as data on age and sex were not available.

### Denmark

Denmark received increasing numbers of Eritrean asylum applications in 2014 (n = 2,275) compared to previous years where maximum annual number was 85. Malaria incidence was 14.5–17.0 per 1,000 Eritreans in 2014 to 2015 and continuously high (43.1 per 1,000 but with wide CI) in 2016. Out of 74 cases during 2014 to 2016 50 were male and the median age during the same period was 24 years. However, only 255 Eritreans sought asylum in Denmark in 2016.

### United Kingdom

The UK received 3,280 and 3,270 asylum applications from Eritrean citizens in 2014 and 2015 respectively. The UK was the country with the highest overall annual number of malaria cases, but the increase was not as pronounced, with 1,501 cases in 2013 and 1,586 cases in 2014 (p = 0.13). This increase was not significant, but country of birth was missing for a large proportion of cases (reported in 4,010/7,537 (53.2%) of cases in 2011–2015). When malaria incidence malaria among Eritreans was calculated, it was lower compared to the other countries studied (6.1, 5.4 and 10.0 /1,000 in the years 2014, 2015 and 2016) with low numbers of registered cases among Eritreans (20 per year in 2014 and 2016 and 13 in 2016). The majority of patients were male (18/20 in both 2015 and 2016), *P. vivax* was the dominating species among Eritreans and age median age in this group was 20 years in 2014 and 21 years in both 2015 and 2016.

## Discussion

This analysis of European data on annual asylum seekers and imported malaria cases revealed a sudden and significant rise in malaria cases in seven European countries during 2014 and 2015. The increase was mainly explained by *P. vivax* cases in Eritreans. The seven countries accounted for 93% of applications from Eritrean asylum seekers in Europe. We found that the increase in malaria cases was not only due to a higher number of asylum applications compared to previous years; there was also a substantial increase in the proportion of Eritreans affected by the disease, especially among young male adults and adolescents. The patients reported migrating from Eritrea through Ethiopia and/or Sudan and Libya before arriving in Europe, and many had repeated febrile episodes on their migration route. The review of medical records in Germany and Sweden found a relatively high proportion of severe *P. vivax* malaria, predominantly characterised by severe anaemia, and more frequently in Sweden compared to Germany. The difference in proportion of severe cases is surprising and could imply that malaria diagnosis was delayed in Sweden, or because the data was collected in two different settings. Importantly, low haemogloblin levels were associated with longer symptom duration.

Our analysis of national surveillance data and medical records is consistent with previous reports of *P. vivax* clusters in Eritrean migrants in Europe in 2014 [4-6] as well as more recent national reports [7-9], and provides a more complete and detailed picture. The estimated mean incidence rate in the seven countries in the study increased from 2 malaria cases per 1,000 Eritrean asylum applications in 2013 to 18 cases per 1,000 in 2014. In 2016, although the number of Eritrean asylum applications remained high in some countries such as Germany, the number of malaria cases in this group dropped markedly, suggesting either changes in the epidemiology of malaria along the route of migration or a possible change in migration routes. We estimated that there were at least 1,500 malaria cases in Europe during 2014 and 2015 that can be attributed to newly arrived Eritreans. However, the number of malaria cases in Eritrean migrants in Europe during this period is likely to be even higher, as an unknown number of arriving Eritreans might have sought medical care in countries along their migration route, as reports from Italy suggest [13]. Part of the increase might be due to greater awareness and increased testing by clinicians or microbiological laboratories in parallel with the greater number of cases that were diagnosed from 2014 onwards. A limitation of this report is that countries were selected based on registered asylum applications from Eritreans and furthermore, detailed data was available only for Germany and Sweden. Nonetheless, the seven EU/EFTA countries receiving 93% of Eritrean asylum applicants contributed data.

Patients in Hamburg (Germany) and Sweden reported migrating from Eritrea through Ethiopia and/or Sudan and Libya before arriving in Europe. According to the WHO World Malaria Report, *P. vivax* accounted for 26% of clinical malaria cases in Eritrea, 41% in Ethiopia and 5% in Sudan in 2014 [14]. Clusters of *P. vivax* malaria cases in Eritreans have previously been reported in Israel and Jordan [15-17]. To our knowledge, there have been no reports published on epidemics or increased *P. vivax* incidence in Eritrea, Ethiopia or Sudan during 2014 and 2015, but rather a decline in transmission has been observed [14]. Libya is considered to be malaria-free, although recent case reports raise suspicion of autochthonous *P. falciparum* and *P. vivax* transmission in the country [18]. The high incidence in Eritrean asylum applicants (higher than in Eritrea) thus suggests some high-level local malaria transmission along their route of migration, possibly in eastern Ethiopia where *P. vivax* transmission is reported to be high [1].

Malaria was predominantly diagnosed in male teenagers with 1 case in 20 male asylum applicants. Lack of acquired immunity in adolescents who have grown up in Eritrea where malaria transmission is low [14] may have contributed to a particularly high level of vulnerability in this group to parasite exposure during migration. Noteworthy, many patients reported that they were already suffering from malaria attacks during migration, and treatment of *P. vivax,* not including radical cure of hypnozoites, may have resulted in relapsing infections. Indeed, analysis of weekly data in Sweden revealed that the *P. vivax* cases were predicted by the number of asylum applications from Eritreans 5 weeks prior to the actual malaria notifications, which may reflect relapses rather than primary infections, in particular considering that most patients had left malaria-endemic areas several months earlier. Stress and generally impaired health status of the host during and immediately after the migration process could be possible contributions to relapses of malaria [19]. Our findings highlight the need to focus and report on active malaria transmission on migration routes and to promote local interventions. Countries receiving migrants from Africa need to be aware that the epidemiology of imported malaria may change rapidly and that relapses of *P. vivax* can occur several months after arrival in Europe.

The geographical patterns of imported malaria in Europe were recently modelled using national statistics [20]. Despite covering the same time period, modelling from aggregated data did not seem to capture the high number of *P. vivax* cases in a particular migrant group. Indeed, using only reported country of infection (especially only one country) has limitations as reflected by our comparison: clinical medical records and notification data showed coherence of the country of infection in only 35% of patients (reflecting limitations both in medical recordings and notification data). If this data can be collected, it could result in better risk assessment and more rapid detection of infections, in particular among vulnerable migrant groups.

Europe was declared malaria-free by WHO in 2016 [21]. Nonetheless, *Anopheles* mosquitoes with competence in malaria transmission are present in large parts of Europe [22]. A proportion of migrants in Sweden and Germany did not attend treatment follow-up and had unclear compliance regarding primaquine. As relapse frequency after treatment is 7–8% this might possibly be a public health issue. The risk of secondary cases in some parts of Europe thus exists but is relatively small; public health professionals need to be aware of the entomology data and which mosquito species are present in their area of responsibility and should develop strategies to successful eradication treatment of *P. vivax* [1,22].

Our review of medical records showed that severe malaria cases and repeated episodes were common. Haemogloblin levels correlated with symptom duration and a high proportion of severe anaemia requiring blood transfusions in Sweden is believed to be a result of prolonged symptoms and/or repeated episodes which are factors known to contribute to vivax anaemia [23]. Healthcare and general conditions are expected to be poor for refugees during migration, and displaced populations have been reported to be at increased risk of malaria [24]. Any febrile patients (including refugees) who have travelled through or arrived from malaria-endemic areas must be thoroughly investigated, including a blood test for malaria. Clinicians and healthcare workers need to be aware of potentially severe symptoms. Screening refugees or asylum seekers arriving from malaria endemic areas could contribute to a more rapid diagnosis and treatment, and migrant populations from other parts of Africa or the Indian subcontinent could be considered. Irrespective of this, it is important to ensure migrants’ access to healthcare, especially for potentially severe diseases, on the basis of the international human rights law regarding migrants’ right to health [25].

In conclusion, we report a remarkably high incidence of vivax malaria among newly arrived Eritrean asylum seekers in Europe during 2014 and 2015. Policy makers as well as individual healthcare workers need to be prepared for rapid changes in the patterns of imported malaria, and possibly other infectious diseases, in migrants and particularly in refugees and asylum seekers who may have undertaken long journeys through several countries to Europe.

## Supplementary Data

416000 Supplementary Figure S1

## Supplementary Data

110000 Supplementary Table S2
