# Supplementary material for: Malaria in Eritrean migrants newly arrived in seven European countries, 2011 to 2016
Source: Euro Surveill. 2019 Jan 31;24(5):1800139. doi: 10.2807/1560-7917.ES.2019.24.5.1800139 (PMC6386211; doi:10.2807/1560-7917.ES.2019.24.5.1800139)
Supplement: Supplementary Figure S1 [file 1800139_SONDEN_SupplementaryFigure.pdf]

This supplementary material is hosted by Eurosurveillance as supporting information alongside the article “Malaria in Eritrean migrants newly arrived in seven European countries, 2011 to 2016” on behalf of authors Sondén et al who remain responsible for the accuracy and appropriateness of the content. The same standards for ethics, copyright, attributions and permissions as for the article apply. Eurosurveillance is not responsible for the maintenance of any links or email addresses provided therein.

Figure

Number of asylum applications per month in Europe 2008-2016; **a)** total (grey line) and Eritrean origin (blue bars); **b)** in Germany; and **c)** in Sweden; and number of imported malaria cases diagnosed in Germany 2008-2016 (green bars)**(d)** and Sweden**(e)**. Each bar represents one month.

a

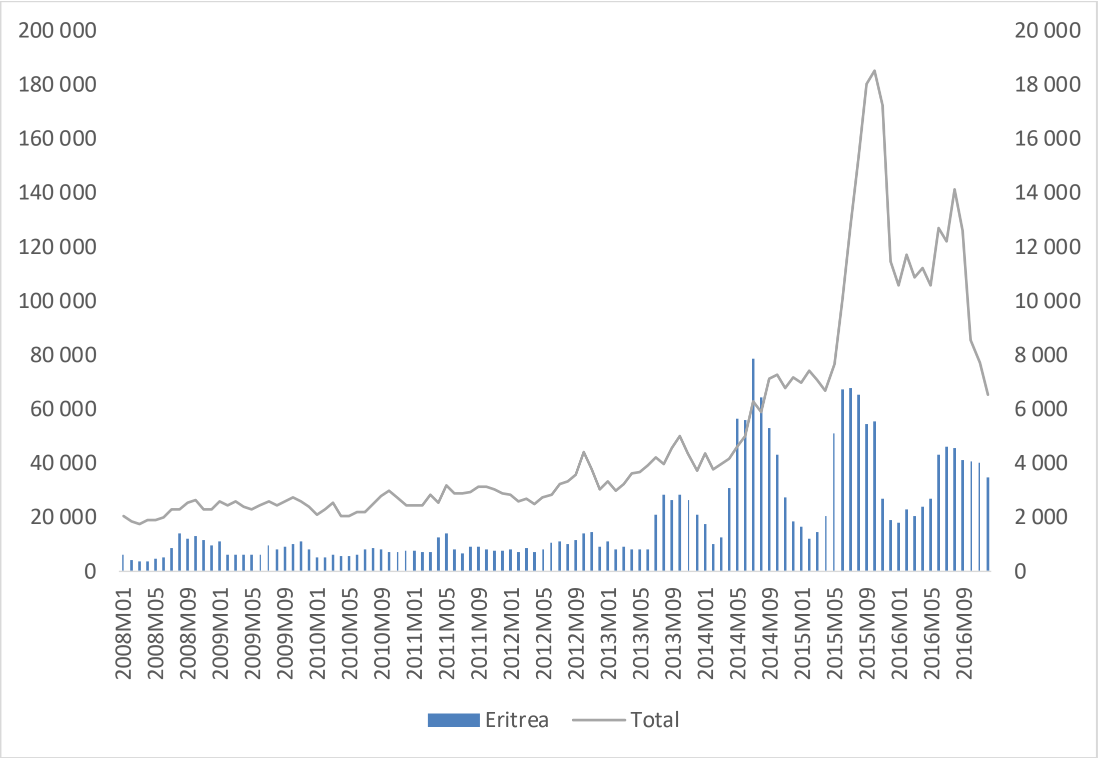
200 000
180 000
160 000
140 000
120 000
100 000
80 000
60 000
40 000
20 000
0
20 000
18 000
16 000
14 000
12 000
10 000
8 000
6 000
4 000
2 000
0
2008M01
2008M05
2008M09
2009M01
2009M05
2009M09
2010M01
2010M05
2010M09
2011M01
2011M05
2011M09
2012M01
2012M05
2012M09
2013M01
2013M05
2013M09
2014M01
2014M05
2014M09
2015M01
2015M05
2015M09
2016M01
2016M05
2016M09
Eritrea
Total

b

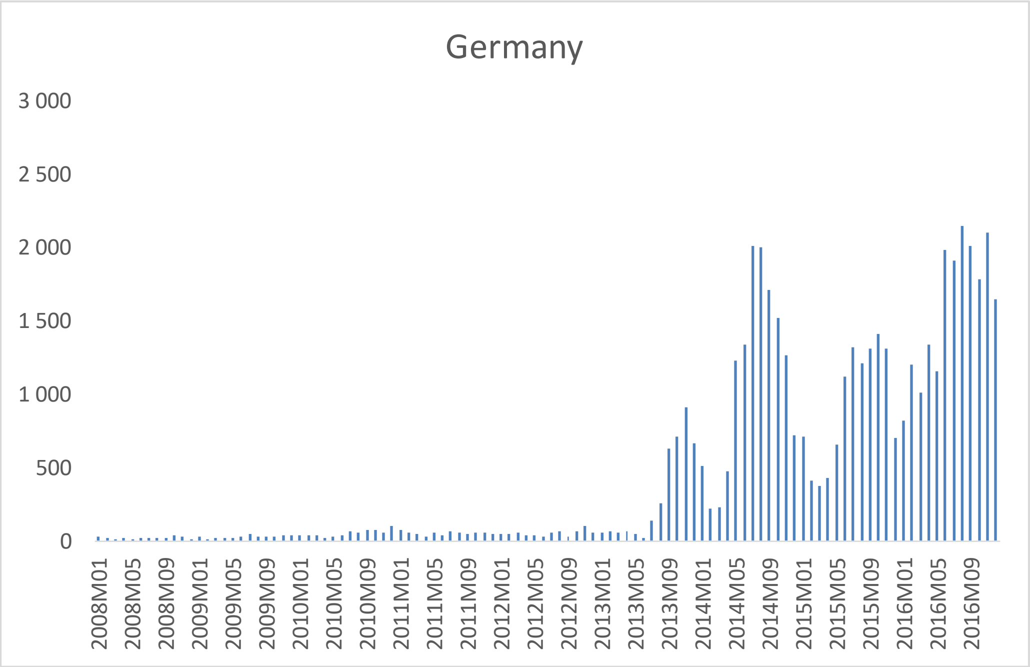
Germany
3 000
2 500
2 000
1 500
1 000
500
0
2008M01
2008M05
2008M09
2009M01
2009M05
2009M09
2010M01
2010M05
2010M09
2011M01
2011M05
2011M09
2012M01
2012M05
2012M09
2013M01
2013M05
2013M09
2014M01
2014M05
2014M09
2015M01
2015M05
2015M09
2016M01
2016M05
2016M09

c

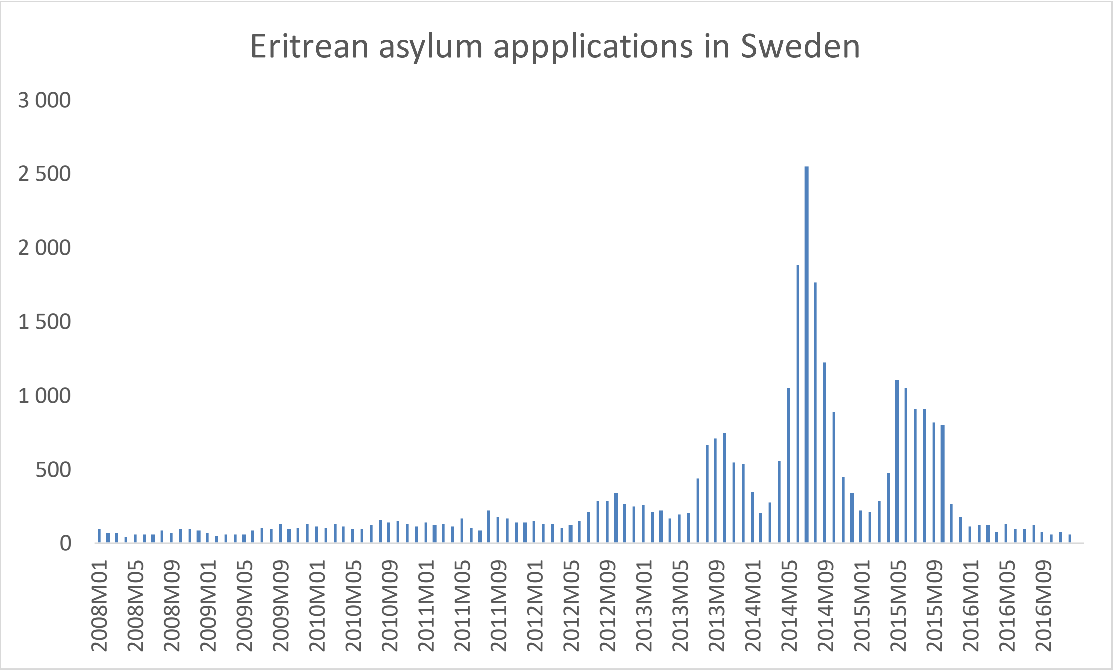
Eritrean asylum appplications in Sweden
3 000
2 500
2 000
1 500
1 000
500
0
2008M01
2008M05
2008M09
2009M01
2009M05
2009M09
2010M01
2010M05
2010M09
2011M01
2011M05
2011M09
2012M01
2012M05
2012M09
2013M01
2013M05
2013M09
2014M01
2014M05
2014M09
2015M01
2015M05
2015M09
2016M01
2016M05
2016M09

d

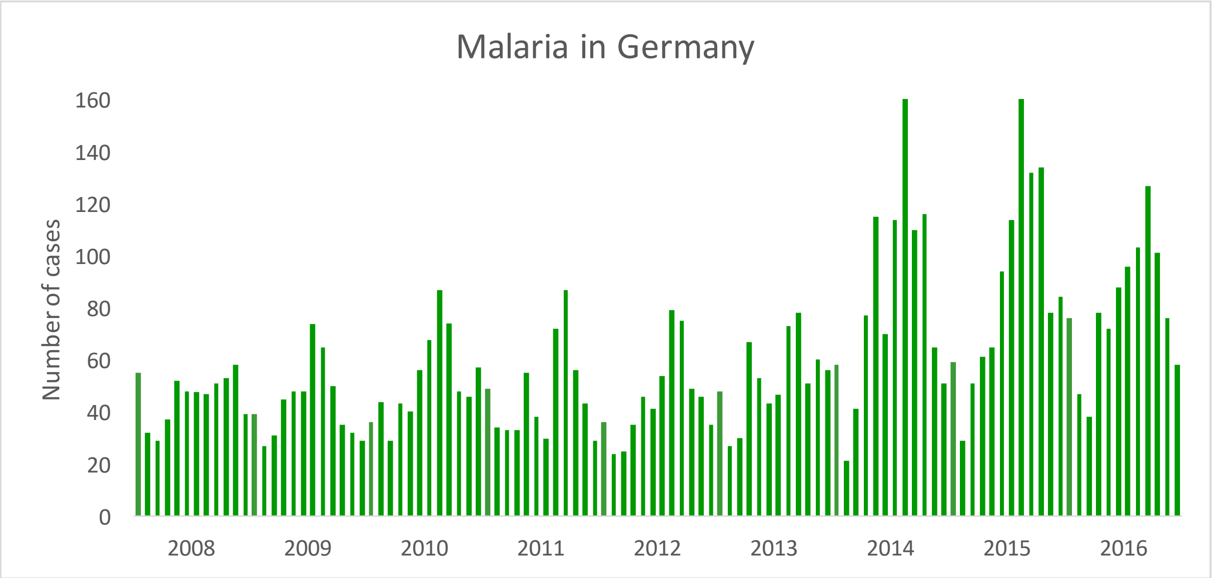
Malaria in Germany
Number of cases
160
140
120
100
80
60
40
20
0
2008
2009
2010
2011
2012
2013
2014
2015
2016

e

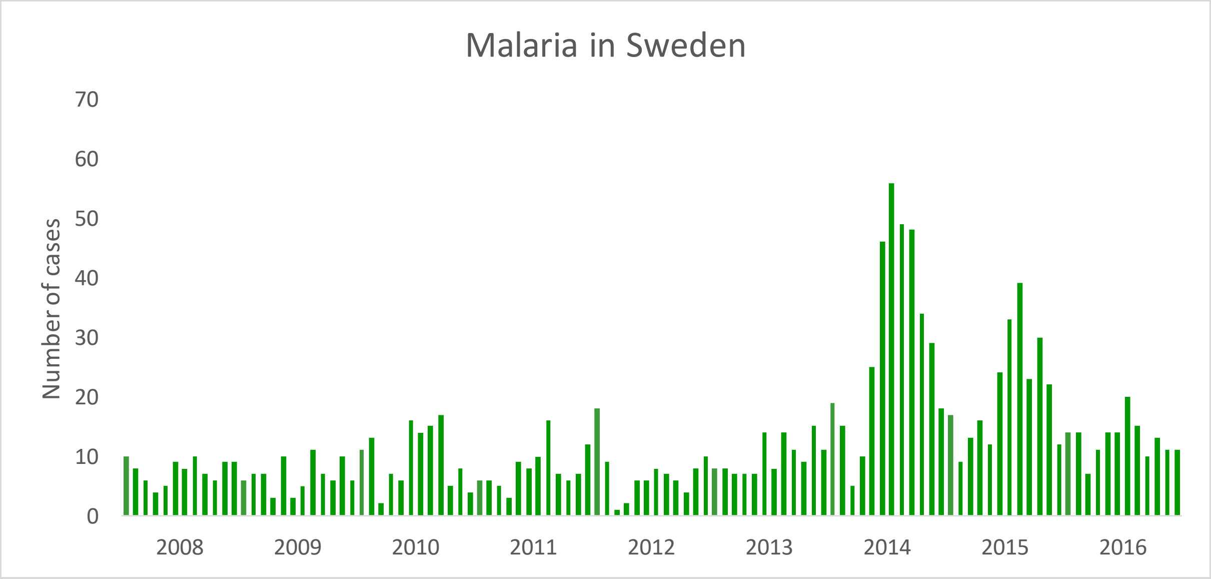

Malaria in Sweden
Number of cases
70
60
50
40
30
20
10
0
2008
2009
2010
2011
2012
2013
2014
2015
2016
