# Supplementary material for: Malaria in Eritrean migrants newly arrived in seven European countries, 2011 to 2016
Source: Euro Surveill. 2019 Jan 31;24(5):1800139. doi: 10.2807/1560-7917.ES.2019.24.5.1800139 (PMC6386211; doi:10.2807/1560-7917.ES.2019.24.5.1800139)
Supplement: Supplementary Table S2 [file 1800139_SONDEN_SupplementaryTable.pdf]

This supplementary material is hosted by Eurosurveillance as supporting information alongside the article “Malaria in Eritrean migrants newly arrived in seven European countries, 2011 to 2016” on behalf of authors Sondén et al who remain responsible for the accuracy and appropriateness of the content. The same standards for ethics, copyright, attributions and permissions as for the article apply. Eurosurveillance is not responsible for the maintenance of any links or email addresses provided therein

Supplementary table 1. Malaria cases in Germany 2011-2015 reporting Eritrea as country of infection and/or Eritrea as country of origin

		2011	2012	2013	2014	2015	p
**Eritrea as country of infection**		**0**	**0**	**3**	**91**	**80**	
**Eritrea as country of origin**		**0**	**0**	**7**	**244**	**181**	
**Country of infection**	**Eritrea**			3	82	66	0.19
	**Ethiopia**			0	6	9	
	**Horn of Africa**			1	9	12	
	**Nigeria**			0	0	1	
	**Northern Africa**			0	1	0	
	**South Sudan**			0	1	0	
	**Sudan**			0	11	2	
	**Missing data**			3	124	91	
**Eritrea as country of origin and/or of infection**		**0**	**0**	**7**	**253**	**195**	
**Sex**	**Male**			7	227	176	1.00
	**Female**			0	22	17	
	**Missing data**			0	4	2	
**Age (years)**	**median**			20.18	20.47	20.10	
	**IQR**			17.09-24.85	17.51-24.43	17.18-23.76	
**Arrival year in Germany**	**1991**			0	1	0	
	**2012**			0	1	1	
	**2013**			1	3	1	
	**2014**			0	155	15	
	**2015**			0	0	104	
	**Missing data**			6	93	74	

**Species**	***P. vivax***	4	198	146	0.82
	P. falciparum	0	12	10	
	**Other**	0	38	33	
	**Missing data**	3	5	6	
